# GenoSurf: metadata driven semantic search system for integrated genomic datasets

**DOI:** 10.1093/database/baz132

**Published:** 2019-12-09

**Authors:** Arif Canakoglu, Anna Bernasconi, Andrea Colombo, Marco Masseroli, Stefano Ceri

**Affiliations:** Dipartimento di Elettronica, Informazione e Bioingegneria, Politecnico di Milano, Piazza Leonardo da Vinci 32, 20133 Milan, Italy

## Abstract

Many valuable resources developed by world-wide research institutions and consortia describe genomic datasets that are both open and available for secondary research, but their metadata search interfaces are heterogeneous, not interoperable and sometimes with very limited capabilities. We implemented GenoSurf, a multi-ontology semantic search system providing access to a consolidated collection of metadata attributes found in the most relevant genomic datasets; values of 10 attributes are semantically enriched by making use of the most suited available ontologies. The user of GenoSurf provides as input the search terms, sets the desired level of ontological enrichment and obtains as output the identity of matching data files at the various sources. Search is facilitated by drop-down lists of matching values; aggregate counts describing resulting files are updated in real time while the search terms are progressively added. In addition to the consolidated attributes, users can perform keyword-based searches on the original (raw) metadata, which are also imported; GenoSurf supports the interplay of attribute-based and keyword-based search through well-defined interfaces. Currently, GenoSurf integrates about 40 million metadata of several major valuable data sources, including three providers of clinical and experimental data (TCGA, ENCODE and Roadmap Epigenomics) and two sources of annotation data (GENCODE and RefSeq); it can be used as a standalone resource for targeting the genomic datasets at their original sources (identified with their accession IDs and URLs), or as part of an integrated query answering system for performing complex queries over genomic regions and metadata.

## 1. Introduction

Next-generation sequencing technologies and data processing pipelines are rapidly providing sequencing data with associated metadata, i.e. high-level features documenting genomic experiments. Very large-scale sequencing projects are emerging, and many consortia provide open access for secondary use to a growing number of such valuable data and corresponding metadata. While the provided sequencing data are generally of high quality and increasingly standardized, metadata of different sources are differently structured; furthermore, their search interfaces are heterogeneous, not interoperable and sometimes with very limited capabilities. However, modern biological and clinical research more and more takes advantage of integrated analysis of different datasets produced at various sources; therefore, a system capable of supporting metadata integration and search, able to locate heterogeneous genomic datasets across sources for their global processing, is strongly needed.

We built such metadata integration and search system; our approach is based on the genomic conceptual model (GCM, (1)), which provides a small set of entities and attributes for metadata description, covering very important and complex data sources. On top of this core schema, we implemented a multi-ontology semantic search system that uses knowledge representation for supporting metadata search. Our metadata repository currently includes about 40 million metadata entries from five sources, out of which more than 7 million have been integrated within the common structure of the GCM, described by 39 attributes over eight connected entities. We also provide semantic enrichment of the values of 10 of these attributes, by linking them to ontological terms. For each of such terms, besides describing synonyms and other syntactic and semantic variants, we provide a small hierarchy of hypernyms and hyponyms, whose depth typically ranges up or down to three hierarchical levels.

Our metadata repository can be searched with a friendly web user interface called GenoSurf, publicly available at http://www.gmql.eu/genosurf/. Through it, the user can: (i) select search values from the integrated attributes, among predefined normalized term values optionally augmented by their synonyms, and hypernyms; (ii) obtain a summary of sources and datasets that provide matching items (i.e. files containing genomic regions with their property values); (iii) examine the selected items’ metadata in a tabular customizable form; (iv) extract the set of matching references (as backlinks to the original sources and links to data and metadata files); (v) explore the raw metadata extracted for each item from its original source, in the form of key-value pairs; (vi) perform free-text search on attributes and values of original metadata; and (vii) prepare data selection queries ready to be used for further processing. Search is facilitated by drop-down lists of matching values; aggregate counts, describing resulting files, are updated in real time.

The metadata content is stored in a PostgreSQL database, including for each item a backlink to the original source storing the referenced data. It is fueled by an automatized pipeline to register new sources and extract their metadata, as well as to update and maintain already integrated sources. The pipeline systematically performs data extraction, translation, normalization and cleaning. Using it, we integrated metadata from five consolidated genomic sources: The Cancer Genome Atlas (TCGA, (2)) from Genomic Data Commons (GDC, (3,4)); The Encyclopedia of DNA Elements (ENCODE, (5,6)); Roadmap Epigenomics (7); GENCODE (8); and RefSeq (9), the latter two providing reference annotation data. Furthermore, we are in the process of adding other data sources, including Cistrome (10), International Cancer Genome Consortium (ICGC, (11)), and 1000 Genomes Project (12), and we plan to integrate several others.

We also imported processed genomic data into an integrated data repository, where they can be globally handled with our high-level, declarative GenoMetric Query Language (GMQL, (13)) (http://www.gmql.eu/gmql-rest/) and associated GMQL repository engine (14), which uses Apache Spark (https://spark.apache.org/) on arbitrary servers and clouds. The data repository currently contains 243 520 files from 37 datasets; repository versions have been available since 2017 (http://www.bioinformatics.deib.polimi.it/GeCo/) and were used in several collaborative (epi) genomic projects. GenoSurf data items are in one-to-one mapping with the most recent version of the data files in the GMQL repository and share the same identifiers. Hence, the result of a GenoSurf search can be immediately used within the GMQL engine (44) to extract and directly process comprehensively relevant genomic region data files and their metadata.

### Related works

In the past few years, several surveys (15–17) have highlighted the need for data integration approaches in life sciences, with a particular attention to omics. Among various projects focused on offering integrated access to biomedical data and knowledge extracted from heterogeneous sources, we cite BioMart (18), DNADigest (19), DATS (20), BioSchemas (21), FAIR (22) and MOD-CO (23)—all single initiative or community-driven efforts towards making biomedical data findable and accessible for scientific datasets.

For what concerns the semantic enrichment of metadata, (24) surveys the use of ontologies in biomedical data management and integration. Choosing the most suitable ontologies for semantic enrichment of specific corpuses of values is addressed in (25).

We consider DeepBlue (26) as the most similar platform to ours. This data server was developed to search, filter and process epigenomic data produced within a multi-center research consortium. Some of its modeling choices are similar to ours (e.g. distinction between region data and metadata, management of both experimental and annotation datasets, a set of mandatory attributes and key-value pairs to store additional metadata). However, DeepBlue focuses on epigenomics, while we approach a broader integration, as we consider a larger spectrum of different data/experiment types. The DeepBlue database identifies five mandatory metadata attributes (three of them are standardized to external controlled vocabularies and equipped with synonyms and hierarchies), while GenoSurf accounts for eight entities with 39 attributes (10 of which are normalized, also including synonyms, hierarchies and external references).

### Paper organization

We first describe the relational database schema on which our repository is based, including the tables that support semantically expanded search (using specialized ontology content). Then, we describe the currently integrated data sources, spanning different areas of functional genomics such as expression and mutation data (TCGA/GDC), epigenomics (ENCODE, Roadmap Epigenomics) and annotations (RefSeq and GENCODE). The main focus of this paper is a thorough description of a novel web server for searching the integrated repository; we show the user interface, explain how inference occurs in the background, describe several use cases and provide an evaluation.

## Relational schema

**Figure 1:**
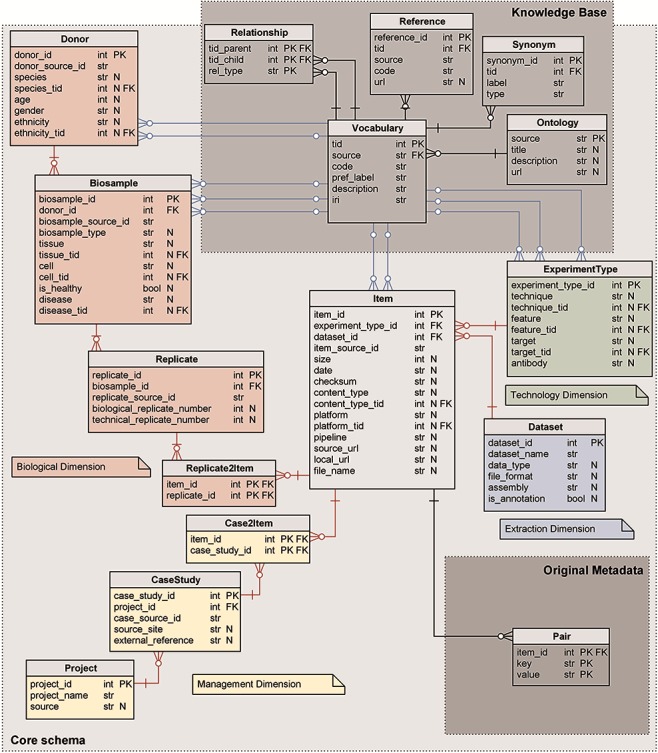
Logical schema of the metadata repository. Red relations represent FKs between core schema tables; blue relations link core schema values to corresponding ontology vocabulary terms. Data types are shortened: str for character varying; int for integer; and bool for Boolean. N marks nullable attributes.

The core of our metadata repository exhibits a star-like relational schema, illustrated in Figure 1, centered on the Item table; it physically implements the GCM (1), as each table corresponds to a GCM entity. The core schema is extended by two subschemas representing, respectively: the original unstructured metadata—in the form of key-value pairs—and the semantic enrichment for specific attributes of four core tables (Knowledge Base).

### Core schema

The core schema is a classic data mart (27), with a central fact table describing Items (or data files) and four dimensions:

- The Biological dimension describes the biological material and process observed in the experiment that generated the genomic item. It includes Donor, Biosample and Replicate entity tables, and the Replicate2Item bridge table.
- The Management dimension describes the organizations or projects that are behind the production of each experiment. It includes the Project and CaseStudy entity tables, and the Case2Item bridge table.
- The Technology dimension describes the process used for the production of the experimental or annotation item and includes the ExperimentType entity table.
- The Extraction dimension describes the containers available in the repository for storing items that are homogeneous for data analysis; it includes the Dataset entity table.

All core tables have a numerical sequential primary key (PK), conventionally named <table_name>_id and indicated as PK in Figure 1. Tables Donor, Biosample, Replicate, Item and CaseStudy have, in addition, a secondary unique key <table_name>_source_id that refers to the original source; such secondary key is used for providing backward links to the data source (and for direct comparison of source contents with the ones in the repository during periodic updates/reloads).

Core tables have two kinds of foreign keys (FKs): the FKs that uniquely identify a row of another table of the core schema (red in Figure 1) and FKs that reference concepts in the Knowledge Base from the core attributes that are semantically enriched (blue in Figure 1). Nullable attributes are indicated in Figure 1 with N.

Relationships in the core schema from the Item outward are functional (i.e. one Item has one ExperimentType, while an ExperimentType may be the same for multiple Items), with the exception of two many-to-many relationships: each Item derives from one or more Replicates and belongs to one or more CaseStudies.

We next discuss every table of the core schema.

#### Item

Each item corresponds to a processed data file that contains genomic region data. It references the ExperimentType and Dataset tables with FKs, whereas item_id is directly used in bridge tables Replicate2Item and Case2Item. Size, date and checksum denote the properties of the corresponding genomic data file: source_url; local_url; file_name; and source_page include information useful to locate and download the physical data file and associated information. The content_type describes the type of genomic regions in the file (such as gene segments, introns, transcripts, etc.) and is enriched using concepts in the NCIT (28) and SO (29) ontologies. The platform is the instrument used to sequence the raw data related to the item and is enriched using the OBI ontology (30). The pipeline includes a list of methods used for processing phases, from raw data to processed data.

#### Replicate

When an assay is performed multiple times on separate biological samples (or even on the same sample), multiple replicas of the same experiment are generated, each associated with a distinct item and progressive numbers (indicated as biological_replicate_number and technical_replicate_number). Multiple replicates for the same item are present in the sources ENCODE and Roadmap Epigenomics.

#### Replicate2Item

This bridge table, by combining item_id and replicate_id, can associate multiple Items to a single Replicate (i.e. they may have undergone different processing) and multiple Replicates to a single Item (such items are generally called ‘combined’).

#### Biosample

It describes the material sample taken from a biological entity and used for the experiment. It references the Donor table with an FK. The biosample_type distinguishes between tissues, cell lines, primary cells, etc. The tissue field is enriched by concepts in the Uberon ontology (31), describing a multicellular component in its natural state, or the provenance tissue of cells. The cell field allows to specify single cells (in natural state), immortalized cell lines or cells differentiated from specific cell types; it is enriched by concepts in the EFO (32) and CL (33) ontologies. The disease (i.e. illness investigated within the sample) is enriched by the NCIT ontology; the is_healthy field stores a Boolean condition, as the biological sample may be healthy/control/normal or non-healthy/tumoral.

#### Donor

It describes the donor providing the biological sample. The donor age, gender, ethnicity (enriched with terms from the NCIT ontology) and species (enriched with terms from the NCBITaxon terminology (34)) refer to the individual from which the biological sample was derived (or the cell line established).

#### CaseStudy

It connects the set of items that are collected together, as they participate to the same research objective (the criteria used by each source to group together such files are variable). It references the Project table with an FK. The source_site represents the physical site where the material is analyzed and experiments are physically produced (e.g. universities, biobanks, hospitals, research centers or just laboratory contact references when a broader characterization is not available). External_reference may contain identifiers taken from the main original source and other sources that contain the same data.

#### Case2Item

This bridge table, by combining item_id and case_study_id, can associate multiple Items to a single case (which is the typical scenario) but also multiple cases to a single Item (this happens when an Item appears in multiple analyses and studies).

#### Project

It represents the infrastructure or organization that sets the context for the experiments (or case studies). Source describes the programs or consortia responsible for the production of genomic items (currently featuring five possibilities: TCGA; ENCODE; Roadmap Epigenomics; RefSeq; and GENCODE). Within a source, items may be produced within a specific initiative, specified in the project_name, which uniquely references the project; it is particularly relevant in the context of TCGA data, where items are organized based on the type of tumor analyzed in the specific project (e.g. BRCA identifies a set of items regarding the Breast Invasive Carcinoma study) or in annotation projects (such as the RefSeq reference genome annotation).

#### ExperimentType

It refers to the specific methods used for producing each experimental or annotation data file (hence, each item of the core schema). With respect to the original source, a tuple is uniquely identified by the triple technique, feature and target. The first one is enriched by the OBI or EFO ontologies and describes the assay, i.e. the investigative procedure conducted to produce the items. The second one is enriched by the NCIT ontology and describes the specific genomic aspect studied with the experiment (e.g. gene expression, mutation and histone mark). Epigenomic experiments such as ChIP-seq usually analyze a protein, which we call target; this field is enriched by concepts in the OGG ontology (35). The antibody is the protein employed against such target (values refer to The Antibody Registry, http://www.antibodyregistry.org/, or the ENCODE antibody accession, in case the first is missing).

#### Dataset

It gathers groups of items stored within a folder named dataset_name; dataset items are homogeneous as they share a specific data_type (e.g. peaks, expression quantifications and methylation levels), assembly (i.e. reference genome alignment—either hg19 or GRCh38) and file_format (i.e. standard data format of the items dictating the genomic region data schema, including the number and semantics of attributes, for example BED, narrowpeak or broadpeak). The Boolean variable is_annotation allows distinguishing between datasets containing experimental data and datasets storing genomic annotations (currently defined in the Item’s content_type field).

### Original metadata

Out of about 40 million metadata extracted from sources, around 7 million were included in the core schema. Many attributes and their respective values found within different sources cannot be mapped to the same conceptual model. We store such extra attributes in an unstructured format, using key-value pairs extended with the item_id of the Item which they refer to; all attributes together form the PK, while the item_id also acts as FK.

### 2. Knowledge base

Some of the attributes of the core schema have been annotated with ontological concepts using an automatic procedure following described. Enriched attributes include: the ethnicity and species describing Donors; disease, tissue and cell describing Biosamples; technique, feature and target describing ExperimentTypes; and platform and content_type describing Items.

Automatic enrichment is performed by using one or two preferred bio-ontologies for each attribute (details on the annotation process are available in (36)). For a given value, when a match with an ontology term is not found, the annotation task is re-routed to a manual procedure handled by an admin user who is expert in data curation and biomedical ontologies. So far, we enriched attribute values by linking them to 1629 terms in the eight specified ontologies. In addition to terms that directly annotate core values (and their synonyms), we included all terms that could be reached by traversing up to three ontology levels from the base term (12 087 concepts in total); as next discussed, the use of three levels enables powerful query extensions.

Also the Knowledge Base is deployed using relational tables; in particular, we use:

- 1) the Vocabulary table, whose PK term identifier tid is referenced from all the core tables that contain semantically enriched attributes, with the acronym of the ontology providing the term (source, e.g. NCIT), the code used for the term in that ontology (e.g. NCIT_C4872) and its label (pref_label, e.g. Breast Carcinoma), in addition to an optional description and iri (i.e. International Resource Identifier);
- 2) the Ontology table, a dimension table presenting details on the specialized ontologies contained (even partially) in the knowledge base—referenced with an FK from the vocabulary table;
- 3) the Reference table, containing references to equivalent terms from other ontologies (in the form of a < source, code> pair)—referencing, with the FK tid, the term in the vocabulary table;
- 4) the Synonym table, containing alternative labels that can be used as synonyms of the preferred label along with their type (e.g. alternative syntax, related nomenclature and related adjectives)—referencing the term in the vocabulary table; and
- 5) the Relationship table, containing ontological hierarchies between terms and the type of the relationships (either generalization is_a or containment part_of)—the PK is composed of parent, child and type of the relationship; the first two reference the vocabulary table with FKs.

For performance issues, we materialized an unfolded representation of the Relationship table and a denormalized representation of the core tables, which are used by search queries; they are rematerialized at each change of the database.

**Figure 2:**
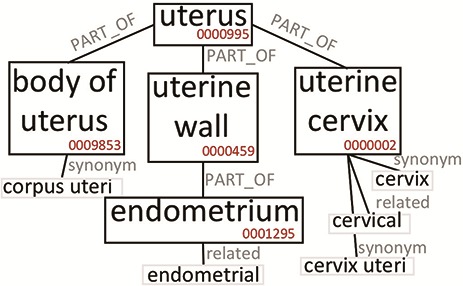
Excerpt of Uberon subtree originating from the ‘uterus’ root. We only report elements that are relevant to our example.

The construction of the Knowledge Base allows to expand the semantic content of the values contained in the GCM. To clarify this aspect, we briefly discuss the case of the broad ‘uterus’ concept. In our system, tissue values are extended using the Uberon ontology, which represents body parts, organs and tissues in a variety of animal species (adopted by many groups and projects, such as the Gene Ontology (37), Monarch Initiative (38), EBI (https://www.ebi.ac.uk/) and ENCODE). Figure 2 is an excerpt of Uberon useful to grasp the ontological structure containing the concepts interesting for this example. The ‘uterus’ concept (ID:0000995) includes, among others, three parts: ‘body of uterus’ (ID:0009853), ‘uterine cervix’ (ID:0000002), and ‘uterine wall’ (ID:0000459). The last one has the ‘endometrium’ part (ID:0001295). Each concept can be related to exact or broad synonyms, related adjectives and alternative syntaxes.

Table 1 reports which values (Search keyword) in our system are mapped to the ‘uterus’ or related ontological concepts, and how many data items are retrieved when the query matching mechanism progressively includes richer options, moving from the original values (Original) up to include also the equivalent values (Synonym) and the hierarchical hyponyms (Expanded). Depending on the used option, the number of matches changes significantly. By using the Original value option, the search for ‘uterus’ retrieves only 57 data items; when the matching includes synonyms, it returns 1708 items (as it also matches ‘uterus nos’, which stands for ‘uterus, not otherwise specified’). When the matching includes hyponyms (Expanded), it returns 16 851 items, as it matches also all other terms in Table 1, which originate from the ‘uterus’ root in Uberon (Figure 2). The user can choose any of the three levels of matching for queries and obtain either exact or semantically improved matches without needing to know the specific nomenclature used in the various integrated data sources.

**Table 1:** Number of items found in GenoSurf using different search options and keywords from the ‘uterus’ concept area

Term ID	Search keyword	Original	Synonym	Expanded
0000995	uterus	57	1708	16 851
	uterus nos	1651	1708	16 851
0009853	body of uterus	0	9535	9535
	corpus uteri	9535	9535	9535
0000002	uterine cervix	0	5585	5585
	cervix uteri	5417	5585	5585
	cervix	167	5585	5585
	cervical	1	5585	5585
0000459	uterine wall	0	0	23
0001295	endometrium	21	23	23
	endometrial	2	23	23

Original refers to search at the core level (such values correspond to metadata retrieved from original sources); in addition to original values, synonym considers also equivalent terms and synonyms, whereas expanded considers also all terms in the ontological subhierarchy.

## 3. **Data sources**

Our system targets genomic sources, in the typical broader meaning that includes DNA data, but also epigenomic and transcriptomic data. However, our framework is general enough to include other kinds of omics data, e.g. for proteomics or metabolomics, which can be described with the few attributes of our core schema.

Data are extracted through an incremental integration framework, realized as a standalone application developed in Scala programming language and configurable through an XML file. The framework extracts both metadata (then accessed by using GenoSurf) and region data (to be imported in the GMQL repository). Our data extraction procedure, which requires human intervention and is supported by software modules, includes six steps.

The download step handles heterogeneity at the distribution and format level, by taking into account various access protocols (FTP, HTTP, RESTful API and file bundles) as well as data formats (XML, JSON, CSV, Excel and Google Sheet); it imports at our repository site the original data and their metadata from the sources, making use of a precise source partitioning scheme to allow for versions’ comparison. A transformation step flattens metadata into a key-value output format, where the attribute (key) describes the kind of represented information and the value embodies the actual information. A cleaning step produces a collection of standard metadata pairs for each source. Redundant information (i.e. duplicated attributes) is removed, and cumbersome keys deriving from the previous phase are filtered out. A mapping step extracts information from the produced pairs and maps it to the core tables (only pairs relevant to the GCM, while others are kept in key-value format). To this end, we manually specify ad hoc mappings between the entities of the core schema and cleaned attributes from the sources; examples of rules are provided in (1). In the enrichment step, the values in specific attributes of the core tables are then linked to biomedical ontologies, typically manually curated by experts. During this step, values that have super-concepts or subconcepts in the biomedical ontologies are enriched with all terms in a is_a or part_of relationship within three steps in the ontology graph, as described in the previous section ‘Knowledge Base’ and detailed in (36, 45). Finally, we perform a content consistency check step to enforce integrity constraints and legal values in the repository. Examples of the applied rules are present in (1).

The whole project is detailed in https://github.com/DEIB-GECO/Metadata-Manager/wiki/ and the code released as open source.

**Figure 3:**
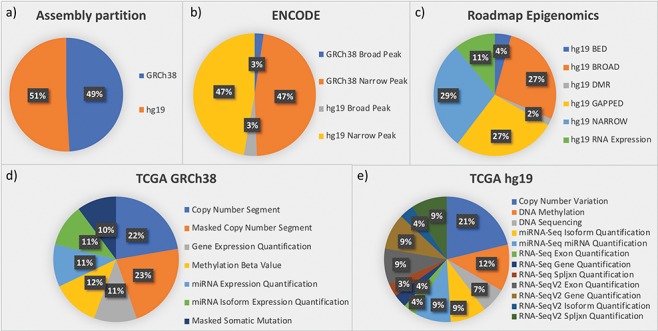
Partition of data in the integrated repository according to (a) assemblies and datasets from the most relevant sources: (b) ENCODE; (c) Roadmap Epigenomics; (d) TCGA in the GRCh38 version provided by GDC; and (e) TCGA in the hg19 legacy data repository.

The framework was designed starting from three important data sources: ENCODE, Roadmap Epigenomics and TCGA. These three sources provided us with the most complex integration scenarios that can be faced in genomic metadata integration. Later, exploiting the generality of the integration framework, we easily extended our repository by adding genomic annotations (from GENCODE and RefSeq). Figure 3 provides quantitative descriptions of the datasets currently in the repository, all related to the human species and roughly equally distributed between assemblies hg19 and GRCh38 (see Figure 3a).

ENCODE collects projects regarding functional DNA sequences that intervene at the protein/RNA levels. From ENCODE, as of July 2019, we have included in our data repository all available processed human data files in narrowpeak and broadpeak format, for both GRCh38 and hg19 assemblies (see four datasets in Figure 3b).

Roadmap Epigenomics is a public resource of human epigenomic data; as indicated in Figure 3c, we included in GenoSurf 6 datasets regarding NGS-based consolidated processed data about: (i) broad and narrow regions associated with protein binding sites, histone modifications or open chromatin areas identified using ChIP-seq or DNase-seq experiments (datasets NARROW, BROAD, GAPPED and BED); (ii) differentially methylated regions (dataset DMR); and (iii) expression quantification of regions associated with genes, exons and other known genomic regions of interest (dataset RNA Expression).

TCGA is the most relevant source for cancer genomics, with data about RNA and miRNA expressions, copy number variations, somatic mutations and methylation levels. In (39), we reported the development of an automatic pipeline to transform into BED format, the data originally available at the former TCGA portal, based on hg19 assembly. The portal is now deprecated and has been replaced by the GDC project (https://gdc.cancer.gov/), which provides data for the GRCh38 assembly; we transformed into BED format, also this updated version of the TCGA data (http://www.bioinformatics.deib.polimi.it/opengdc/). As to July 2019, we imported seven GRCh38 datasets with a total of 100 234 data files (Figure 3d) and 12 hg19 datasets with a total of 106 780 data files (Figure 3e).

GENCODE aims at creating a comprehensive set of annotations, including genes, transcripts, exons, protein-coding and non-coding loci, as well as variants. For the hg19 assembly, we included releases 10 and 19, whereas for GRCh38, we imported versions 22, 24 and 27.

RefSeq is a stable reference for genome annotation, analysis of mutations and studies on gene expression. We imported annotation files of GRCh38 v10 and hg19 v13 releases.

## 4. **Web interface**

Users can search the integrated metadata content through the GenoSurf web interface, which allows to specify search values for metadata attributes and retrieve a list of matching genomic items (i.e. corresponding genomic data files).

**Figure 4:**
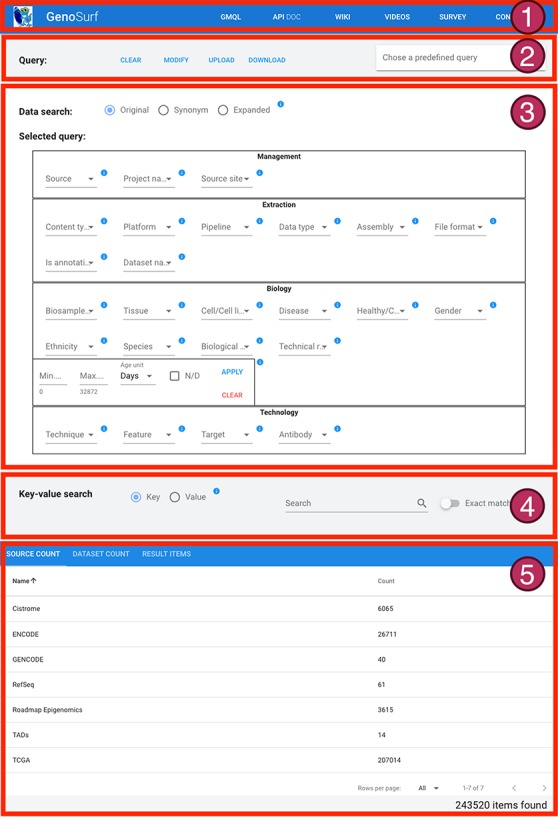
Sections of GenoSurf Web interface: (i) top menu bar; (ii) query utilities; (iii) data search; (iv) key-value search; and (v) results visualization.

The interface is composed of five sections, described in Figure 4: (i) a menu bar to navigate the different services and their documentation; (ii) intuitive query utilities; (iii) the search interface over the core database, whose content can be set on three levels: original metadata, synonyms/alternative syntax and hierarchical ontological expansion; (iv) the search interface over key-value pairs, for searching over original metadata from the imported sources; and (v) a result visualization section, showing the resulting items in three different aggregation sections. The interface also enables an interplay between core Data search and Key-value search, thereby allowing to build complex queries given as the logical conjunction of a sequence of core metadata and key-value search steps of arbitrary length; results are updated at each step to reflect the additional search conditions, and the counts are dynamically displayed to help users in assessing if query results match their intents. In the following, we describe the main GenoSurf sections more in detail.

### 5. Simple data search

The Data search section (part 3 of Figure 4) serves as a primary tool for querying the integrated repository with GenoSurf. To improve usability, it is based on a restricted number of the attributes in the repository schema; attribute names in the interface are slightly changed with respect to relational table fields, with the purpose of facilitating their understanding. The interface allows setting different levels of semantic enrichment: the Original option (search using metadata values provided by original data sources), the Synonym option (adding synonyms) and the Expanded option (adding hypernyms and hyponyms).

The Data search section has four parts to reflect the four dimensions of GCM, i.e. Management, Extraction, Biology and Technology. It contains the 26 attributes of the core tables most relevant for search purposes (other core attributes are managed as additional key-value pairs). For each attribute, matching values are presented for selection in a drop-down list; each value has on the side the number of items connected in the star schema to that value. Multiple values chosen for the same attribute are considered as possible alternatives (in disjunction); values chosen over different attributes are considered as conditions that should all be satisfied (in conjunction) by the resulting items. The special value N/D indicates null values and allows to select items for which a particular attribute is undefined. After each selection, a running query is progressively built and shown in the interface field ‘Selected query’; the current query is evaluated, and the number of matching items is displayed.

**Figure 5:**
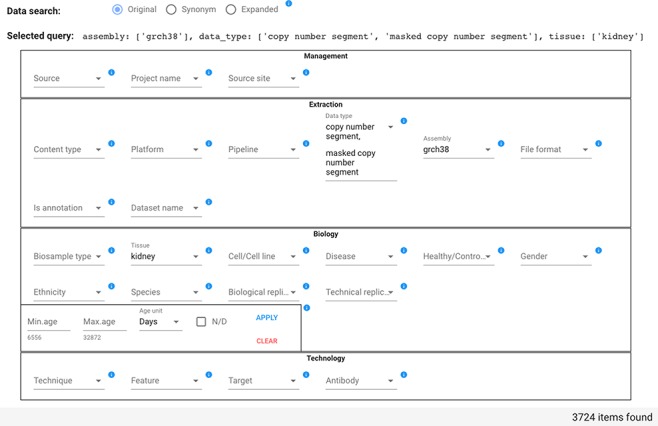
Data search section of the GenoSurf Web interface, highlighting attributes within the four dimensions of the repository core schema; values are entered by users and appear in drop-down menus for easing their selection.

In the example shown in Figure 5, the user searches for all items that have Data type either ‘copy number segment’ or ‘masked copy number segment’ and that have Assembly ‘grch38’ and Tissue ‘kidney’; the query option is set to Original. As a consequence of the attribute value selection, the field ‘Selected query’ is compiled as:

assembly: [grch38], data_type: [copy number segment, masked copy number segment], tissue: [kidney].

Counts of attribute value associated items are changed dynamically. For example, if initially the Data type drop-down menu shows 22 371 items for ‘copy number segment’ and 22 374 for ‘masked copy number segment’, when the user selects the Tissue ‘kidney’ (with count 19 357), then the Data type drop-down menu shows 1862 items for each of the two mentioned Data types, reflecting the reduction of matching items.

As an example of query with Data search option set to Synonym, when we only select the value ‘k562’ for the Cell/Cell line attribute (which at the Original level had a 5942 count), we obtain a count of 5986 items, which is the same for all equivalent syntactic variants of the attribute (e.g. ‘k-562’, ‘k562 cell’ and ‘k-562 cell’). Indeed, the additional 44 items derive from a small set of items labeled with ‘k562 leukemia cells’, which have been annotated with such synonym concept corresponding to the term EFO_0002067 in the Experimental Factor Ontology. As a second example, assuming we are interested in the antibody Target BORIS (Brother of Regulator of Imprinted Sites), at the Original level, we cannot find any match in the repository. However, when we enable the Synonym level search, we find 10 items (which were originally annotated with the transcriptional repressor CTCFL), since in the Ontology of Genes and Genomes the concept OGG_3000140690, with preferred label CTCFL, has the alternative term BORIS.

As an example of Expanded search, if we select the value ‘eye’ for Tissue, we find 1473 items by exploiting the expansion offered by the Uberon ontology. Specifically, we retrieve: 13 items annotated exactly with ‘eye’; 1440 items annotated with ‘Eye and adnexa’ (all from TCGA), which is an alternative form of ‘eye’; and also 20 ENCODE items annotated with ‘retina’, which *is_a* ‘photoreceptor array’, which is in turn a *part_of* ‘eye’.

### 6. Key-value search

The Key-value search section (part 4 of Figure 4) allows searching metadata without having previous knowledge of the original metadata attribute names and values, or of the attribute names and data content of the GCM core schema, which stands behind the integration effort. In the Key-value search, the user can perform a case-insensitive search either over all metadata attributes (using the Key option) or over all metadata values (using the Value option). Users can search both keys and values that either exactly match or only contain the input string.

**Figure 6:**
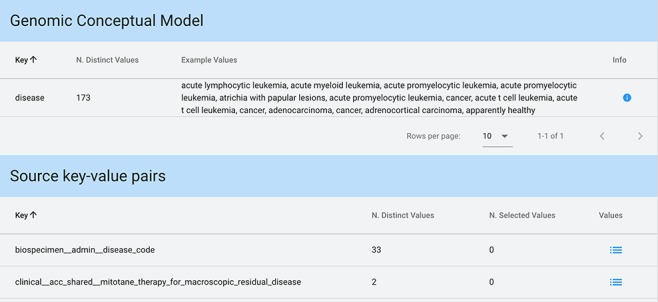
Key-value search result using input string ‘disease’ as a key. The keyword is matched both in the GCM attributes (for each matching attribute, we present the number of available distinct values and some example values) and in the original source attributes (each matching attribute enables exploration and selection of any corresponding values).

When input strings are searched within keys, in case a match is found among the core attributes of the GCM (which can also be considered as ‘keys’), we provide an informative result: example values of each of the matched attributes and the number of distinct values available for that attribute. Conversely, when showing the results of a match on original attributes (i.e. keys), a list of all matching keys is provided in output, equipped with the number of distinct values available for each of such keys; the user can then explore these values and select any of them. Figure 6 shows a search with Key option and input string ‘disease’.

Value search has a simpler interface, showing all possible matches in values, both for core attributes and original key-value pairs. Users can directly select desired key-value pairs among the ones shown in the result.

### 7. Query sessions

**Figure 7:**
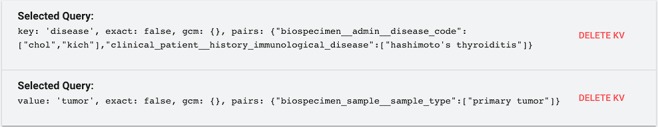
Example of composition of two key-value search sessions.

A query consists of a sequence of search sessions, performed by alternating simple Data search and Key-value search; a sequence of searches produces items resulting from the conjunction of search conditions. Within each search session, multiple options for values (either for core attributes or as keys/values in Key-value search) are considered in disjunction. Figure 7 shows how a query can be composed using a sequence of two Key-value search sessions; steps can be deleted by rolling them back in any order. The query of Figure 7 corresponds to the predicate:

(biospecimen__admin__disease_code = chol OR biospecimen__admin__disease_code=kich OR clinical_patient__history_immunological_disease = hashimotos thyroiditis) AND.

biospecimen_sample__sample_type = primary tumor.

Every choice in the Data search and Key-value search sections impacts the results and their visualization at the bottom of the web interface page (part 5 of Figure 4). Acting on the Data search section, the result table is updated whenever the user either adds or removes a value from a drop-down menu or types/deletes text directly in a text field. In the Key-value search section, filters are instead applied/deleted by pressing corresponding buttons; due to their greater complexity, they are typically applied one-by-one, hence a dynamic update is not useful.

### 8. Result visualization

**Figure 8:**
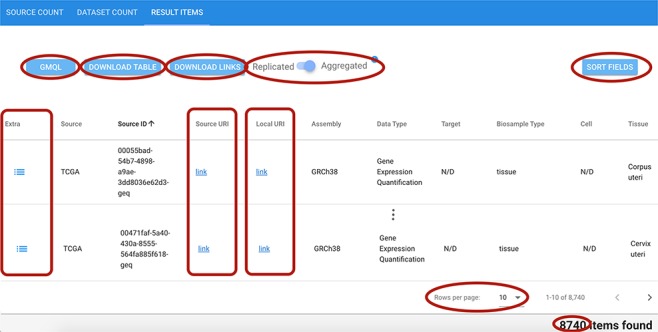
Excerpt of the result items table resulting from a search session. Red ellipses highlight relevant features. Top left: GMQL button to generate queries to further process related data files; DOWNLOAD buttons for result items table and data file links; and Replicated/Aggregated switch. Top right: SORT FIELDS button to customize the columns visualized in the table. Center: Extra, Source URI and Local URI columns with clickable links. Bottom right: component to set the number of rows visible at a time; indication of the total items corresponding to the performed query.

As shown in Figure 8, the result visualization includes three sections: (i) Source count, containing the number of found items aggregated by origin data source; (ii) Dataset count, containing the number of found items aggregated by dataset name; and (iii) Result items, reporting the core metadata values of resulting items (to be navigated in batches of chosen cardinality, with suitable scroll options). Within the last section, a table is presented with one row for each item; for all found items, we also provide links to the data description page at the source location, and to data files at their source location (Source URI) and at our repository location (Local URI). The user can visualize the original metadata key-value pairs of a data file by clicking on the row’s Extra button. In the bottom part of the table, the user can select how many rows should be visible in the page, up to a 1000 limit; other pages can be scrolled using the left/right arrows. Fields can be arbitrarily sorted, included or excluded from the tabular visualization (Sort fields button).

The user can change the one-item-per-row default view by using the Replicated/Aggregated switch; when items match many Replicates/Biosamples/Donors, with the Aggregated option, related information is aggregated by concatenating the possible distinct values through the pipe symbol ‘|’.

### 9. Inference explanation

Behind the scenes, the implemented keyword-based search is driven by a precise inference mechanism, which is tuned according to the user’s choice of query option (Original, Synonym and Expanded) and is based on semantic enrichment. To illustrate the relational links that are traversed in the different kinds of searches over our system, we introduce the concept of deduction chain, which describes the internal path in the database that links the Item (i.e. the found data file) to the table where the match with the search keyword is found. The deduction chain shows the steps of the inference process that are activated according to the requested search level. A search may be performed considering:

- the source original metadata key-value pairs;
- the GCM attributes;
- additionally, the ontological synonym annotations; and
- additionally, the ontological hierarchical expansions.

The best way to illustrate deductive chains is by showing an example of use. A biologist or bioinformatician may be interested in the keyword ‘brain’, intending to request all data items related to this concept (i.e. those that contain this string in their metadata). Table 2 shows how quantitative results in our system (i.e. numbers of items) can be explained: according to the different search levels (first column); we indicate the number of found data files (second column); and the third column shows the deduction chain.

**Table 2:** Available search levels and examples of their results for the ‘brain’ search keyword

Search level	# items	Deduction chain
1	789	<Item> − <Key: biosample__organ_slims, Value: brain>
	4670	<Item> − <Key: gdc__project__disease_type, Value: brain lower grade glioma>
	126	<Item> − <Key: clinical__lgg__family_history_of_primary_brain_tumor, Value: yes>
	2463	<Item> − <Key: clinical_patient__history_lgg_dx_of_brain_tissue, Value: no>
2	15 714	<Item> − <Replicate> − <Biosample.tissue: brain>
	9188	<Item> − <Replicate> − <Biosample.disease: brain>
	10	<Item> − <Replicate> − <Biosample.cell: smooth muscle cell of the brain vasculature>
3	13	<Item> − <Replicate> − <Biosample.cell: fetal brain> − <Vocabulary: brain, UBERON_0000955>
4	10	<Item> − <Replicate> − <Biosample.tissue: pons> − <Vocabulary: pons, UBERON_0000988 > −[IS_A] − < Vocabulary: regional part of brain, UBERON_0002616 > −[PART_OF] − < Vocabulary: brain, UBERON_0000955>
	8	<Item> − <Replicate> − <Biosample.tissue: globus pallidus> − <Vocabulary: globus pallidus, UBERON_0001875 > −[PART_OF]− < Vocabulary: pallidum, UBERON_0006514 > −[IS_A]− < Vocabulary: brain gray matter, UBERON_0003528 > −[PART_OF]− < Vocabulary: brain, UBERON_0000955>

For the different search levels, we show the number of items resulting from searching on: (1) source key-value metadata; (2) core attribute values; (3) synonyms; and (4) hyponyms, with the corresponding deduction chain.

At the first level, the search produces key-value pairs corresponding to unchanged original metadata directly linked to the <Item>. Thus, the first four rows of Table 2 link an <Item> directly to a < Key, Value>; at this level, term matching can be performed on either keys or values. For instance, the first row indicates that 789 found items are associated with the pair <biosample__organ_slims, brain>.

At the second level, the search is performed on the attributes of the core schema; results in Table 2 match values contained either in the tissue, disease or cell attributes. In the first case, the deduction chain shows that 15 714 items are found since they are connected to a < Replicate>, further connected to a < Biosample>, which contains the ‘brain’ value for the tissue attribute.

At the third level, the search is based on ontological vocabularies and synonyms. The example in Table 2 shows that we found 13 items whose original cell value is ‘fetal brain’, a synonym of ‘brain’ annotated with the Uberon ontology term 0000955.

At the fourth level, the search is based on ontological vocabularies and synonyms or their hyponyms; for instance, the first row in Table 2 for this search level indicates that 10 items are associated with the term ‘pons’, which is a ‘regional part of brain’ according to the Uberon ontology terms 0000988 and 0002616.

### Additional functionalities

To provide a complete and useful environment to users, we allow to modify, save and load queries, as well as search results, in a customizable way; other functionalities allow to use results produced by GenoSurf within the GMQL engine.

#### Interaction with queries

To support re-use of queries, we provide the possibility to download and upload text files in JavaScript Object Notation (JSON) format containing the query, or directly copy, paste and modify JSON queries on the web interface. Furthermore, 10 predefined queries are available to demonstrate practical uses of the interface.

#### Use of results

Found genomic region data files can be downloaded individually from the GenoSurf web interface using the Source URI, a clickable link to download the region data file from the origin source, or the Local URI, to download the region data file corresponding to the selected item from the GMQL system, when available (see Figure 8). Additionally, for each search query, we provide (through the buttons DOWNLOAD TABLE and DOWNLOAD LINKS in Figure 8): a text file containing all the URLs to download all the genomic region data files from our system, and a comma-separated file to download the entire results table.

Finally, the user can generate a GMQL query (button GMQL in Figure 8) that can be used directly in our GMQL engine in order to select specifically the items found with a GenoSurf search for further processing.

#### RESTful API

All services used in the GenoSurf web interface are implemented using our GenoSurf RESTful Application Programming Interface (API) available at http://www.bioinformatics.deib.polimi.it/genosurf/api/.

All POST services are based on the principle of setting a JSON payload that establishes the context for the next query. As an example, if the JSON payload is as follows: {gcm:{disease:[prostate adenocarcinoma], assembly:[grch38]}, type:riginal, kv:{} }, it means that the next query (i.e. API request) is performed only on the set of GRCh38 prostate adenocarcinoma items, which are 4821. Suppose we are interested in knowing how many of these samples are healthy and how many are non-healthy. We can thus call the /field/{field_name} API service (with {field_name} equal to is_healthy) providing the just mentioned payload. The output is as follows:

{

 values: [

  {value: false, count: 3543},

  {value: true, count: 1278}

 ],

 info: {

  shown_count: 2,

  total_count: 2,

  item_count: 4821

 }

}

It indicates that roughly 75% of the results regard tumor samples and about 25% healthy samples.

Complete dumps of the whole database with timestamps are available at http://www.bioinformatics.deib.polimi.it/genosurf/dump/.

## Use cases

In this section, we show typical data retrieval queries performed by a hypothetical user of GenoSurf to select interesting subsets of the integrated repository. More examples of interest can be found in the GenoSurf WIKI page at http://www.gmql.eu/genosurf/.

### Extracting cancer patient data

**Figure 9:**
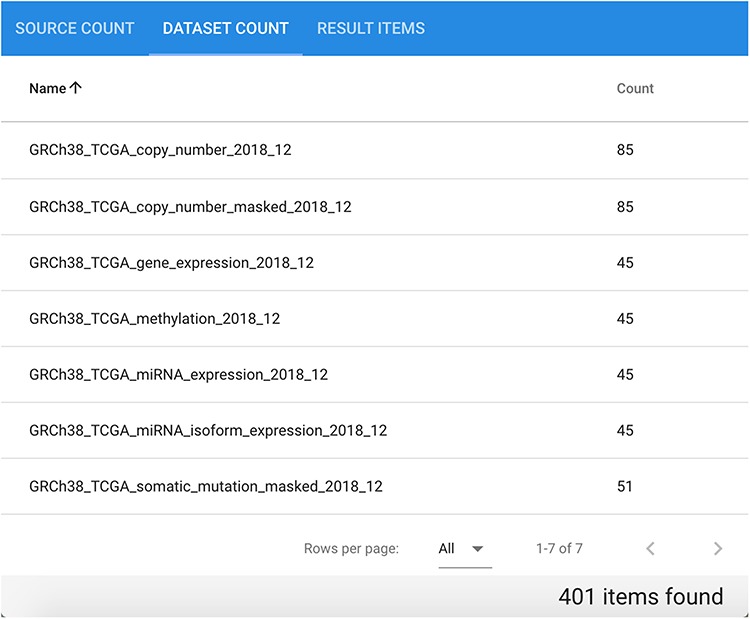
Available datasets for the performed GRCh38 TCGA Cholangiocarcinoma data search.

Suppose we are interested in extracting data of different types divided by patient for a specific cancer type. Considering genomic, epigenomic and transcriptomic data of cancer patients in a comprehensive way provides a general view of their biomolecular system, possibly leading to novel findings. Let us consider as an example GRCh38 TCGA data for disease ‘Cholangiocarcinoma’. In total, the repository contains 401 related items divided in seven datasets, each of which contains between 45 and 85 different items, as it can be observed in Figure 9.

In the RESULT ITEMS table, the order of columns can be customized. For this particular query, it is useful to arrange Source ID, Donor ID, Data Type and Healthy as first columns. The resulting table can be sorted by Donor ID and downloaded as a.csv file. Groups of rows with the same Donor ID represent all available genomic region data files for each specific donor, with different data types and normal/tumor characterization. Table 3 shows an excerpt of the result, relative to two different patients having 9 and 14 items each. The first patient has normal/tumor data pairs for the Copy Number Segment and Masked Copy Number Segment data types, while the second patient has normal/tumor data for all available data types except for Copy Number Segment and Masked Somatic Mutation (in some cases, normal data are even repeated). This kind of quick data extraction can be conveniently used to understand how many same-patient data items are available for performing differential data analysis (i.e. comparison between certain characteristics of normal vs. tumor patients’ signals and sequences).

**Table 3:** Excerpt of result table from the extraction of GRCh38 TCGA Cholangiocarcinoma data, grouped by patient (i.e. Donor ID)

Source ID	Donor ID	Data type	Healthy	Technique
3787a...	07755...	Copy number segment	FALSE	Genotyping array
e6443...	07755...	Copy number segment	TRUE	Genotyping array
f36ef...	07755...	Gene expression quantification	FALSE	RNA-Seq
f7b6d...	07755...	Isoform expression quantification	FALSE	miRNA-Seq
3787a...	07755...	Masked copy number segment	FALSE	Genotyping array
e6443...	07755...	Masked copy number segment	TRUE	Genotyping array
9aa16...	07755...	Masked somatic mutation	FALSE	WXS
1ba92...	07755...	Methylation beta value	FALSE	Methylation array
f7b6d...	07755...	miRNA expression quantification	FALSE	miRNA-Seq
2cbc6...	20bf7...	Copy number segment	TRUE	Genotyping array
c2d57...	20bf7...	Copy number segment	TRUE	Genotyping array
d3b1d...	20bf7...	Gene expression quantification	FALSE	RNA-Seq
2649a...	20bf7...	Gene expression quantification	TRUE	RNA-Seq
016fd...	20bf7...	Isoform expression quantification	FALSE	miRNA-Seq
f002e...	20bf7...	Isoform expression quantification	TRUE	miRNA-Seq
5150...	20bf7...	Masked copy number segment	FALSE	Genotyping array
2cbc6...	20bf7...	Masked copy number segment	TRUE	Genotyping array
c2d57...	20bf7...	Masked copy number segment	TRUE	Genotyping array
80 052...	20bf7...	Masked somatic mutation	FALSE	WXS
33 585...	20bf7...	Methylation beta value	FALSE	Methylation array
d8106...	20bf7...	Methylation beta value	TRUE	Methylation array
016fd...	20bf7...	miRNA expression quantification	FALSE	miRNA-Seq
f002e...	20bf7...	miRNA expression quantification	TRUE	miRNA-Seq

The datasets can be analyzed using a genomic data analysis tool such as GMQL. By clicking on the GMQL button (Figure 8), the user can retrieve the selection query ready to be pasted into the GMQL web interface publicly available at http://www.gmql.eu/gmql-rest/; there, results can be aggregated by patient using specific operations such as JOIN or GROUP BY. For more details, please refer to (14) and to the ‘GMQL introduction to the language’ document at http://www.bioinformatics.deib.polimi.it/genomic_computing/GMQLsystem/documentation.html.

### Combining ChIP-seq and DNase-seq data in different formats and sources

Suppose the data analysis goal is to extract genomic regions of enriched binding sites that occur in open chromatin regions, e.g. focusing on H1 embryonic stem cells. This example shows how to improve the quality of the peaks called within ChIP-seq experiments by filtering out the peaks that are not in open chromatin regions (as required by molecular biology). In order to catch all possible available data related to such cells, we select the Synonym semantic option in the Data search phase. As a first step, we look for ENCODE (Source) and ChIP-seq (Technique) experiment items with narrowpeak format (File format) and regarding H1 cells (Cell/Cell line). We find 601 items as a result. As a second step, we select Roadmap Epigenomics (Source), DNase-seq (Technique) and HOTSPOT (Pipeline) open chromatin regions in H1 cells (Cell/Cell line). Such selection produces as a result four items. For this set, we decide to further restrict the selection to items with a false discovery rate (FDR) threshold of at least 0.01 (note that the HOTSPOT peak caller was used to call domains of chromatin accessibility both with an FDR of 1% and without applying any threshold). Since this is a source-specific metadata information, we apply this filter by using the Key-value search interface: we first search metadata keys that contain the ‘FDR’ string, obtaining the manually_curated__fdr_threshold key with values ‘0.01’ and ‘none’; we then chose to apply the manually_curated__fdr_threshold = 0.01 filter, which reduces our results to only one item, with the desired content. The obtained JSON query corresponding to this second step looks as follows:

{

 gcm:{

  source:[roadmap epigenomics],

  technique:[dnase-seq],

  pipeline:[hotspot],

  cell:[h1 cells]

}

type:synonym,

kv:{

 fdr_0:{

  type_query:key,

  exact:false,

  query:{

   gcm:{},

   pairs:{

    manually_curated__fdr_threshold: [0.01]

    }

   }

  }

 }

}

Such JSON document can be retrieved by pressing the MODIFY or DOWNLOAD query buttons (at the top of the GenoSurf web interface) and can also be used as a payload in the RESTful API services.

The located data files can be either downloaded to be further processed or directly selected in GMQL. Indeed, the objective of this use case corresponds to performing a JOIN operation in GMQL between the regions in the data items found with the first step and those in the item from the second step.

### Extracting Triple-Negative Breast Cancer cases

Suppose we are working on comparative triple-negative breast cancer analysis. This means that we need to select breast tissue data from the TCGA-BRCA project characterized by the absence of all the three types of receptors known to fuel most breast cancer growth: estrogen, progesterone and HER2. Such absence can be encoded in the data as a negative status of the receptors. To do so, first, in the GenoSurf Data search section, we select: project_name: [tcga-brca] and tissue: [breast], which reduces the result to 23 581 items. Then, in the Key-value search section, we need to set the following conditions in conjunction:

clinical__brca_shared__breast_carcinoma_ estrogen_receptor_status:[negative] AND

clinical__brca_shared__breast_carcinoma_ progesterone_receptor_status:[negative] AND

clinical__brca_shared__lab_proc_her2_neu_ immunohistochemistry_receptor_status: [negative]

Note that the exact name of the keys/attributes to query can be identified by previously performing a Key search for estrogen, progesterone or HER2, respectively.

**Figure 10:**
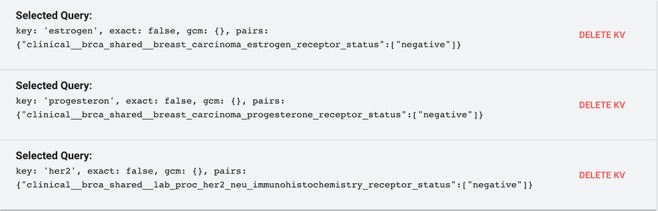
Example of the key-value filters needed to select triple-negative breast cancer items after using the data search interface to preliminarily select TCGA-BRCA breast items.

Figure 10 shows such search on the GenoSurf Key-value interface, leading to the desired result. (Note that for building conjunctive conditions, each one must be in a separate panel; filters selected in the same panel are result in a disjunction).

### Extracting from multiple sources at a time

Suppose we need to retrieve items of hg19 assembly from healthy brain tissue (and possibly its subparts) of male individuals up to 30 years old. In total, hg19 items in the repository are 123 965. Healthy tissue corresponds to choosing ‘true’ in the Healhty/Control/Normal filter, which reduces the result to 18 090 items. Since Tissue is an attribute that benefits from ontological expansion, we select the Expanded semantic option, to be able to find items connected also to the hyponyms of ‘brain’. This filter selects 1046 items (annotated with ‘brain’ or ‘cerebellum’). Gender ‘male’ gets 604 items, and finally, the condition Max.age = 30 years (corresponding to 10 950 days in the API call performed by the system) finds 56 items. As it can be observed in the SOURCE COUNT tab, such output derives from the ENCODE (2 items) and TCGA (54 items) sources.

### Combining mutation and ChIP-seq data

Suppose we are interested in identifying DNA promotorial regions bound by the MYC transcription factor and that present somatic mutations in breast cancer patients with tumor recurrence. To answer such typical biological question, a user can concentrate on hg19 assembly and perform three separate search sessions: (i) selection of ENCODE (Source), hg19 (Assembly), ChIP-seq (Technique), narrowPeak (File format), MCF-7 (Cell/Cell line)—a breast cancer cell line, and MYC binding sites (Target); (ii) selection of TCGA (Source), hg19 (Assembly), BRCA (Project name) and DNA-seq data (Technique) of patients who encountered a new tumor occurrence—such latter information can be selected from the Key-value search part, for example using the value search string ‘new tumor’; and (iii) selection of hg19 genomic region annotations describing promoter locations from RefSeq.

The first result set amounts to 16 items; these can be retrieved by using the filters in the GenoSurf Data search section. The second result set contains three items (first, 993 items are extracted in the Data search section; then, they are reduced to three items by the Key-value search of the additional tumor event). The third result set contains two annotation items, one specific for each assembly.

Such results can be later analyzed in GMQL by using a chain of GenoMetric JOINs first between the sets resulting from selection to extract the MYC-binding promoters, and then with the set from selection, and to extract the BRCA-mutated MYC-binding promoters. Along the way, GMQL operators can be used to remove genomic regions replicated in the data and add new metadata attributes counting the number of investigated promoters for each patient.

## Evaluation

Evaluating an integrated platform for the retrieval of genomic data files is a challenging task. We invited about 60 users, expert of the domain but typically at their first encounter with GenoSurf, to evaluate several aspects of our platform (46). We received 40 complete responses; these helped us to assess the usability and usefulness of our system.

The users were sourced from within our research group (GeCo) at Politecnico di Milano and from several collaborating Institutions (such as Politecnico di Torino, Istituto Nazionale dei Tumori, Università di Torino, Università di Roma Tre, Istituto Italiano di Tecnologia, Radboud Universiteit Nijmegen, Freie Universität Berlin, Harvard University, Broad Institute, National University of Singapore and University of Toronto), including computational and molecular biologists, bioinformaticians and computer scientists/software developers with interest in genomics. To measure the level of experience in the genomic field, each user was asked to rate his/her knowledge in a Likert scale (40) of five levels, from ‘Fundamental Awareness (basic knowledge)’ to ‘Expert (recognized authority)’; users generally self-evaluated to have an intermediate to advanced knowledge, as they use platforms at least on a monthly basis to retrieve and combine data from heterogeneous sources for their analysis. We provided WIKI documentation and video tutorials; half users found this material very useful, while the remaining half did not use it.

Users provided their feedback to a survey (available at http://www.bioinformatics.deib.polimi.it/genosurf/survey/) that includes a set of 10 questions of increasing complexity, performed in order to progressively learn GenoSurf, followed by a questionnaire for providing an overall evaluation of the system; the questionnaire was normally provided in anonymous format, although some users elected to provide their contacts. Correct answers could be read after submitting results.

### Search questions

The 10 questions of the questionnaire (some of which contained two or three subquestions) covered different aspects of the search experience provided by the GenoSurf web interface. We attempted to lower the ambiguity of the questions as much as possible. Starting from the basic selection of data sources and assemblies, we asked the user to explore healthy/tumor options, disease characterizations and data types (e.g. ‘Which TCGA GRCh38 project (among a list of shown options) has more gene expression data?’). We then tested the understanding of the semantic enrichment options (e.g. ‘How many sources contain data annotated with the human fetal lung cell line IMR-90, using alternative syntaxes?’) and the logical combination of different filters (e.g. ‘In ENCODE, how many items of ChIP-seq can you find for the histone modifications H3K4me1, H3K4me2, and H3K4me3?’). One question used the source key-value pair selection, while the final use case proposed the composition of three different datasets to prepare a realistic data analysis scenario (e.g. ‘Suppose you need to identify DNA promotorial regions bound by the MYC transcription factor that present somatic mutations (if any) in breast cancer patients...’), which involved selecting items in separate search sessions from three different data sources.

Five users were able to answer correctly all questions. Overall, eight questions were answered correctly by at least 75% of users. Only two questions had a lower rate of correct answers (respectively, 51.25 and 67.50%). Users responded the 10 test questions in 44 min on average.

### Evaluation of the system

In the second part of the survey, we asked the users whether they liked the system, if they learnt from it, what they suggested to improve and to give us hints on how to proceed in our work (with open suggestions). Two-thirds of the users declared that answering the proposed questions was ‘moderately easy’ or ‘neither easy nor difficult’ and would recommend the platform to their colleagues. Almost all users, when asked to perform a query to reach items useful to their own research, succeeded in their purpose. Most open suggestions proposed the inclusion of currently unsupported sources that we should add, with most indications pointing to the GWAS Catalog, the Genotype-Tissue Expression (GTEx) project, 1000 Genomes Project, the Cancer Cell Line Encyclopedia and the ICGC. A few suggestions indicated small improvements of the interaction that have been incorporated.

## Conclusion

Modern biological and clinical research increasingly takes advantage of high-quality open data, made available by large international consortia. However, the repositories available for data access do not share a common organization for their metadata, and hence, scientists have hard time in searching information from them; a system capable of supporting metadata integration and search, able to locate heterogeneous genomic datasets across sources for their global processing, is strongly needed. We responded to such need by building a metadata integration and search system; the technical development of the integrated repository, its GenoSurf interface and a complete pipeline for metadata acquisition and enrichment started in 2017, and it is now completed.

The repository has been developed using a PostgreSQL implementation; its schema includes core, original and knowledge base data tables, suitably connected by external keys; a materialization of their full join augmented with the unfolding of the knowledge base is also available for fast query processing. The data management backend has been produced in the last 2 years, capitalizing on several previous years of experience in the use of bio-ontologies for specific research projects (e.g. SOS-GeM (41) and GPKB (42)). The repository currently integrates about 40 million metadata items from five sources, described by 39 attributes over eight connected tables of the core schema and enriched with terms from eight different ontologies, which have been reduced to the same knowledge schema.

This metadata search server has been running in the last 6 months; its GenoSurf interface is equipped with RESTful API, documentation Wiki, video tutorials and a pedagogical survey, which are intended to accelerate the users’ learning of the system and its usage. The system has been tested with about 1000 input queries for locating, across sources, heterogeneous datasets targeted to research purposes. They have been generated by the authors, by PhD students and postdocs within the GeCo group, and by about 20 collaborators outside the group, holding either computer science, bioinformatics or biomedical background. External evaluation has been performed on both functional and usability requirements, with positive results summarized by analyzing the answers to a survey filled by 40 researchers with computer science or biomedical background, and with intermediate or advanced expertise on genomics and bioinformatics.

In our future work, we will continue the data integration effort, by completing metadata management for Cistrome and ICGC, as well as 1000 Genomes Project data sources. We will then consider some other sources pointed out by evaluators, starting with the Cancer Cell Line Encyclopedia (43).

From a technological point of view, our next challenge is to design and develop a fast query processing tool and associated RegionViewer, integrated with GenoSurf but directly operating on the GMQL Engine, that can reveal, for given regions or genome bins, individual or summarized information of genetic and epigenetic nature, extracted from processed data in our repository. Such effort will require online fast access to the regions of the repository as well as support for region aggregation operations, hence an efficient redesign of some specific GMQL operators.

## Author Contributions

A.Ca. and A.B. conceptualized the invention and led the design and the evaluation. A.Ca. led the development and implementation of the software. A.B. performed the testing and prepared documentation/use cases. A.Co. contributed to the implementation. S.C. and M.M. conceived and supervised the project. A.Ca., A.B., M.M. and S.C. contributed to the manuscript. All authors read and approved the final manuscript.

## Notes

Database URL: http://www.gmql.eu/genosurf/
